# Supplementary material for: Dynamic Alterations in Acetylation and Modulation of Histone Deacetylase Expression Evident in the Dentine–Pulp Complex during Dentinogenesis
Source: Int J Mol Sci. 2024 Jun 14;25(12):6569. doi: 10.3390/ijms25126569 (PMC11203584; doi:10.3390/ijms25126569)
Supplement: Supplementary file 1 [file ijms-25-06569-s001.zip › ijms-3029053-supplementary.pdf]

Supplementary Table S1. Primer sequences for qRT-PCR

GENE	Forward Sequence (5'->3')	Reverse Sequence (5'->3')	Accession	Primer length (bp)	Product length (bp)
HDAC1 - RAT	ACTTACGAGACAGCTGTGGC	GGAAGGGCTGATGTGAAGCT	NM_001025409.1	20	108
HDAC2 - RAT	TTTTGTCAGCTCTCCACCGG	TCGAGGACAGCAAGCACAAT	NM_053447.1	20	161
HDAC3 - RAT	ACACCCGATGAAACCCCATC	AACCCTGCATATTGGTGGGG	NM_053448.1	20	179
HDAC4 - RAT	TGGAAGAGCTGCAGACAGTG	CTATCCACCCCAACACCACC	NM_053449.1	20	136
HDAC5 - RAT	CCTCAACCATTCCCTCCCAC	TCATAGGGCCCAAGCAAAGG	NM_053450.1	20	135
HDAC6 - RAT	CCGTGAAGGTGCCAACTTTG	AACCTCTCCTGAAAGCACGG	XM_006256756.3	20	115
BETA ACTIN -RAT	CTTCCAGCCTTCCTTCCTGG	AATGCCTGGGTACATGGTGG	NM_031144.3	20	145

Supplementary Table S2 Histone Modifications analysed by mass spectrometry. AC = acetylation; ME = Methylation; UB = Ubiquitination; UN = Unmodified**.** *Several H2A isoforms potentially contribute to the signal for modifications on H2A.**H3R2me2 and H3K4me2/3 are mutually exclusive modifications (i.e. H3R2 methylation prevents H3K4 methylation). As a result, H3K4 modifications are reported only on the H3R2 unmodified peptide (Guccione *et al*. 2007). ♦H3.2 may contribute to signals for modifications labelled H3.1.

**Histone Modifications Detected by Mass Spectrometry**		
H1.4: K25UN	H1.4: K25AC	H1.4: K25ME1
H1.4: K25ME2	H1.4: K25ME3	*H2A: K5UN
*H2A: K5AC	*H2A: K9UN	*H2A: K9AC
*H2A: K36UN	*H2A: K36AC	*H2A1: K13UN
*H2A1: K13AC	*H2A1: K15UN	*H2A1: K15AC
*H2A1: K15UB	*H2A3: K13UN	*H2A3: K13AC
*H2A3: K15UN	*H2A3: K15AC	*H2A3: K15UB
**H3R2UN: K4UN	**H3R2UN: K4AC	**H3R2UN: K4ME1
**H3R2UN: K4ME2	**H3R2UN: K4ME3	H3R2UN: Q5UN
H3R2UN: Q5ME1	H3: K9UN	H3: K9AC
H3: K9ME1	H3: K9ME2	H3: K9ME3
H3: K14UN	H3: K14AC	H3: K18UN
H3: K18AC	H3: K18ME1	H3: Q19UN
H3: Q19ME1	H3: K23UN	H3: K23AC
H3: K23ME1	H3: R42UN	H3: R42ME2
H3: R49UN	H3: R49ME2	H3: Q55UN
H3: Q55ME1	H3: K56UN	H3: K56AC
H3: K56ME1	H3: K64UN	H3: K64AC
H3: K79UN	H3: K79AC	H3: K79ME1
H3: K79ME2	H3: K79ME3	H3: K122UN
H3: K122AC	♦H3.1: K27UN	♦H3.1: K27AC
♦H3.1: K27ME1	♦H3.1: K27ME2	♦H3.1: K27ME3
♦H3.1: K36UN	♦H3.1: K36AC	♦H3.1: K36ME1
♦H3.1: K36ME2	♦H3.1: K36ME3	H3.3: K27UN
H3.3: K27AC	H3.3: K27M	H3.3: K27ME1
H3.3: K27ME2	H3.3: K27ME3	H3.3: K36UN
H3.3: K36AC	H3.3: K36ME1	H3.3: K36ME2
H3.3: K36ME3	H4: K5UN	H4: K5AC
H4: K8UN	H4: K8AC	H4: K12UN
H4: K12AC	H4: K16UN	H4: K16AC
H4: K20UN	H4: K20AC	H4: K20ME1
H4: K20ME2	H4: K20ME3	

References

Guccione, E.; Bassi, C.; Casadio, F.; Martinato, F.; Cesaroni, M.; Schuchlautz, H.; Lüscher, B.; Amati, B. Methylation of histone H3R2 by PRMT6 and H3K4 by an MLL complex are mutually exclusive. *Nature*. **2007**, 449, 933-937. doi: 10.1038/nature06166.
